# The effect of different sterilization methods on the shelf life and physicochemical indicators of fermented pork jerky

**DOI:** 10.3389/fnut.2023.1240749

**Published:** 2023-10-20

**Authors:** Changqing Zhao, Jinping Dai, Feifei Chen, Zhifeng Zhao, Xingxiu Zhao

**Affiliations:** ^1^College of Bioengineering, Sichuan University of Science and Engineering, Yibin, China; ^2^College of Biomass Science and Engineering, Sichuan University, Chengdu, China

**Keywords:** fermented pork jerky, sterilization methods, microbial counts, shelf life, TBARS

## Abstract

**Background:**

To determine the effect of different sterilization methods on shelf life and physicochemical parameters of fermented pork jerky.

**Methods:**

Various sterilization techniques, including boiling, pasteurization, medium-temperature steam sterilization, high-temperature steam sterilization, ultrasonic sterilization, and ultraviolet sterilization, were employed in this study to treat vacuum-sealed fermented pork jerky. Changes in microbial populations, physicochemical parameters, and sensory evaluations were monitored throughout the storage period.

**Results:**

The results indicated the presence of Staphylococcus aureus on the 24th, 21st, 33rd, 24th, 18th, and 15th days in pork jerky subjected to boiling (100°C, 20 min), pasteurization (85°C, 15 min), medium-temperature steam sterilization (105°C, 0.5 Pa, 30 min), high-temperature steam sterilization (121°C, 1.0 Pa, 20 min), ultrasonic sterilization (480 W, 30 kHz, 30 min), and ultraviolet sterilization (254 nm, 100 W/m2, 60 min), respectively. Coliforms, salmonella, and Shigella were not detected in any group during storage. The medium-temperature steam sterilization method yielded the most favorable microbiological results, with an aerobic plate count of less than 1.0 lg CFU/g. However, other physicochemical parameters and sensory evaluations were moderate, with total volatile basic nitrogen (TVB-N) and thiobarbituric acid reactive substances (TBARS) measuring 14.023 mg N/100 g and 0.427 mg MDA/kg, respectively, remaining within acceptable limits.

**Conclusion:**

Therefore, considering microbiological indicators as the primary determinant of shelf life and taking into account other physicochemical parameters, the medium-temperature steam sterilization method was identified as the most suitable approach for extending the shelf life of fermented pork jerky while preserving its flavor.

## 1. Introduction

Meat fermentation stands as one of the oldest and most widely practiced forms of fermentation. It entails a sequence of biochemical, microbiological, and chemical transformations that bestow distinct flavors, colors, and aromas upon fermented meat products. Lactic acid bacteria not only reduce the pH of meat products and generate bacteriocins but also curb the proliferation of pathogens and spoilage microorganisms, thereby augmenting the safety, stability, and shelf life of fermented meat products (1). In our prior studies (2–4), we observed that the sensory attributes of fermented pork jerky surpassed those of non-fermented pork jerky. Furthermore, fermented pork jerky products were packaged using food-grade vacuum sealing. This packaging technique optimizes the preservation of the product’s texture and taste under anaerobic conditions, consequently prolonging its shelf life.

Throughout the production and processing of fermented pork jerky, numerous factors come into play that affect its shelf life. These factors encompass raw materials, workshop hygiene, processing equipment, and personnel. Consequently, in order to ensure product safety and prolong its shelf life, measures such as sterilization (5, 6) are implemented to eliminate contaminating microorganisms. Sterilization methods are primarily categorized into thermal and non-thermal sterilization techniques (7). With technological advancements, innovative sterilization methods have emerged, aiming to preserve the shelf life of products while also considering their taste and flavor. These methods include high-pressure steam sterilization, ultrasonic sterilization, and microwave sterilization. While ensuring sterilization and enzyme inactivation, these techniques also strive to retain the original color, flavor, and nutritional value of food (8), thus markedly enhancing food quality (9). Wang et al. (10) investigated the impact of different sterilization conditions on processed meat products and observed that low-temperature sterilization resulted in improved texture with minimal effects on product structure. Conversely, high-temperature sterilization significantly diminished the texture characteristics of products and exerted some influence on sensory quality. Additionally, microbial indicators during the storage of meat products serve as direct indicators of the product’s shelf life, making them a paramount concern in the industrial production of meat products.

In this study, we selected vacuum-packaged fermented pork jerky as the research subject. We investigated the impact of different sterilization methods, including boiling, pasteurization, medium-temperature steam sterilization, high-temperature steam sterilization, ultrasonic sterilization, and ultraviolet sterilization, on microbial indicators (total aerobic count, total coliform count, Salmonella, *Staphylococcus aureus*, and Shigella), physicochemical indicators [thiobarbituric acid reactive substances (TBARS), total volatile basic nitrogen (TVB-N), pH, and water activity], as well as sensory scores during the storage of fermented pork jerky. Microbial indicators were given primary consideration as they directly reflect shelf life, while other physicochemical indicators were also considered to determine the most suitable sterilization method for fermented pork jerky.

## 2. Materials and methods

### 2.1. Experimental materials and equipment

Fresh pork, white sugar, salt, rapeseed oil, soy sauce, ginger, high-temperature cooking bags, and other ingredients were purchased from the Local supermarkets in Yibin City, Sichuan Province, China. Glucose, lactose, beef extract, peptone, and sodium chloride were purchased from Chengdu Kelong Chemicals (Chengdu, China) and Shanghai Sinopharm Chemical Reagent Co. Ltd. (Shanghai, China).

SS-85 Meat Slicer (WU Qu Industry & Trade Co., LTD). HH-6 Digital display Constant temperature Water bath (Shanghai Lichen Bangxi Instrument Technology Co., LTD). AX124ZH/E Electronic Balance (Ohaus Instrument (Changzhou) Co., LTD). SW-CJ-2F aseptic ultra-clean table (Shanghai Lichen Bangxi Instrument Technology Co., LTD). GZX-9146MBE Electric Heating Constant Temperature Blowing Drying Oven (Shanghai Boxun Industrial Co., LTD.), SHP-80 Biochemical Incubator (Changzhou Putian Instrument Manufacturing Co., LTD). PHS-25 Digital Acidity Meter (Chengdu Fangzhou Technology Development Co., LTD.), SHZ-82 Constant temperature Culture oscillation box (Shanghai Lichen Bangxi Instrument Technology Co., LTD.), DZQ400/2E Vacuum packaging machine (Wenzhou Pentium Machinery Co., LTD.), GI54DWS Vertical automatic Pressure steam sterilizer (Zhiwei (Xiamen) Instrument Co., LTD.), M150B Biological microscope (Shanghai Titan Technology Co., LTD.), K9840 automatic Kjeldahl nitrogen determination instrument (Shandong Haineng Scientific Instrument Co., LTD.), V-1000 Visible Spectrophotometer (Aoyi Instrument Co., LTD.), and HD-3A Moisture activity measuring instrument (Wuxi Huake Instrument Co., LTD.).

### 2.2. Experimental method

#### 2.2.1. Preparing fermented pork jerky

The specific preparation process for fermented pork jerky followed Chen’s method (2). The fermentation of pork jerky occurred under the following conditions: *Lactobacillus delbrueckii* spp. bulgaricus was inoculated at 10%, fermentation lasted for 60 h at 30°C, followed by baking at 95°C for 85 min. Subsequently, the product was sealed in food-grade vacuum packaging with a single-layer thickness of 0.2 millimeters.

#### 2.2.2. Selection of sterilization methods

The vacuum-packed fermented pork jerky underwent sterilization using six different methods, with the specific conditions for these six sterilization methods detailed in Table 1. The sterilized pork jerky is stored in a biochemical incubator at a temperature of 30 degrees Celsius.

**Table 1:** Different sterilization methods (11).

Sterilization method	Sterilization intensity	Sterilization time
Boiling method	100°C	20 min
Pasteurization method	85°C	15 min
Medium temperature steam sterilization method	105°C /0.5 Pa	30 min
High temperature steam sterilization method	121°C /1.0 Pa	20 min
Ultraviolet sterilization method	480 w/30 kHz/40°C	60 min
Ultrasonic sterilization method	254 nm/100(W/m^2^)/22°C	30 min

#### 2.2.3. Microbial parameters detection

In accordance with Chinese national standards (GB 4789.2–2016 (12) for aerobic plate count determination, GB 4789.3–2016 (13) for coliforms count determination, GB 4789.3–2016 (14) for *S. aureus* detection, GB 4789.5–2012 (15) for Shigella detection, and GB 4789.4–2016 (16) for Salmonella detection), analyses were conducted to determine the levels of aerobic plate count, coliforms count, *S. aureus*, Shigella, and Salmonella in fermented pork jerky. The fermented pork jerky, treated with different sterilization methods, underwent testing every 3 days, with each sample weighing 5.0 grams.

#### 2.2.4. TVB-N parameters detection

Based on the automatic Kjeldahl nitrogen analyzer method mentioned in Yang’s study (17), the result was determined as the arithmetic average of three independent determinations obtained under repetitive conditions, with three significant figures retained. The fermented pork jerky, treated with different sterilization methods, underwent testing every 3 days, with each sample weighing 10.0 grams.

#### 2.2.5. TBARS parameters detection

The experimental results were presented based on the spectrophotometry method described in Botsoglou, N. A.’s study (18), representing the arithmetic average of two independent determinations obtained under repeatable conditions, and the results were rounded to three significant figures. Fermented pork jerky treated with different sterilization methods was tested every 3 days, with each sample weighing 5.00 grams. The experiment was conducted three times.

#### 2.2.6. pH parameters detection

A 5-gram sample of minced fermented pork jerky was placed into an Erlenmeyer flask containing 45 milliliters of distilled water. After stirring, the mixture was allowed to stand for 30 min and then was filtered (19). Subsequently, the pH of the filtrate was measured using a precision pH meter (model pHS-25, Chengdu Century Ark Technology Co., Ltd.). The fermented pork jerky treated with different sterilization methods was assessed every 3 days. The experiment was conducted in triplicate.

#### 2.2.7. Aw parameters detection

A water activity meter (HD-3A, Wuxi Huake Instrument Co., Ltd., China) was used to measure the water activity in fermented pork jerky (20). Fermented pork jerky treated with different sterilization methods was tested every 3 days, with each sample weighing 1.00 grams. This experiment was conducted in triplicate.

#### 2.2.8. Sensory evaluation parameters detection

Sensory evaluation of the fermented pork jerky was conducted every 3 days during storage by an experienced sensory panel consisting of 10 members. Eight training sessions were conducted to acquaint the judges with the attributes to assess and the corresponding rating scale. The acceptability of odor, taste, color, and tissue morphology of the fermented pork jerky was evaluated using a 100-point scale as presented in Table 2 (21). The panel sessions were scheduled for mid-morning, taking place in a sensory panel room at a temperature of 22°C. In each session, fermented pork jerky samples from six distinct treatment methods were sequentially assessed. Three slices of each treatment were provided. The order of the samples was randomized within each session, and room-temperature water and unsalted bread were supplied between consecutive samples.

**Table 2:** Criteria for sensory scoring of fermented pork jerky (21).

Sensory attribute	Scoring criterion	Score values
Odor (20 points)	1. Strong meat flavor with unique fermented flavor	15–20
	2. Fermented meat tastes light and unique	10–14
	3. No fermented fragrance, slightly peculiar or stimulating	Under 10 points
Taste (40 points)	1. Soft sour, non-irritating, moderately salty and sweet	35–40
	2. The sour taste is pure, the salty taste is not harmonious, the aftertaste is different.	30–34
	3. Too sour or sour is not obvious, sweet–sour is not harmonious, aftertaste is different	Under 30 points
Tissue (20 points)	1. Partially fractured muscle fiber loose tissue, forming	15–20
	2. Integral muscle fiber dense tissue, forming	10–14
	3. Completely broken muscle fiber loose tissue, unformed	Under 10 points
Color (20 points)	1. Dark red color, uniform, no mildew	15–20
	2. Brown color, uniform, no mildew	10–14
	3. Black brown color, uneven, no mildew	Under 10 points

### 2.3. Statistical analysis

The data underwent statistical analysis using one-way analysis of variance (ANOVA), and the means were distinguished using Tukey’s method at a significance level of 5%. The data analyses were performed utilizing IBM SPSS Statistics 22, a statistical software.

## 3. Results and discussion

### 3.1. Microbial counts during storage of fermented pork jerky prepared by different sterilization methods

In accordance with the Chinese national standard, the aerobic plate count in fermented pork jerky must not exceed 5.0 lg CFU/g, and the coliform count must not exceed 2.0 lg CFU/g. Additionally, pathogens such as *S. aureus*, Shigella, and Salmonella must be absent. In (EC) No 2073/2005, titled “Microbiological Criteria for Foodstuffs,” specific microbiological standards are outlined for pork products. These standards mandate that the total aerobic bacterial count should not exceed 5 × 10^5 CFU/g (Colony Forming Units per gram), and the total coliform count should not surpass 5,000 CFU/g (m3). Furthermore, the presence of pathogens such as *S. aureus*, Shigella, and Salmonella is prohibited. In our experiment, we primarily focused on the aerobic plate count and the presence of *S. aureus*, as no coliforms, Shigella, or Salmonella were detected in any of the groups. Table 3 reveals that *S. aureus* was detected in fermented pork jerky subjected to sterilization methods including boiling, pasteurization, medium-temperature steam, high-temperature steam, ultraviolet, and ultrasonic on the 24th, 21st, 33rd, 24th, 18th, and 15th days of storage, correspondingly. As a result, the shelf life for each group was 21 days, 18 days, 30 days, 21 days, 15 days, and 12 days, respectively.

**Table 3:** The aerobic plate count and *S. aureus* during storage of pork jerky treated by different sterilization methods (lg CFU/g).

Storage time (days)	Sterilization method					
	Boiling method	Pasteurization method	Medium temperature steam sterilization method	High temperature steam sterilization method	Ultraviolet sterilization method	Ultrasonic sterilization method
0	<1.0^c^ +	2.54 ± 0.17^b^ +	<1.0^c^ +	<1.0^c^ +	4.39 ± 0.03^a^ +	4.7 ± 0.06^a^ +
3	2.56 ± 0.06^b^ +	2.56 ± 0.33^b^ +	1.62 ± 0.26^c^ +	1.97 ± 0.02^c^ +	4.59 ± 0.02^a^ +	4.79 ± 0.01^a^ +
6	<1.0^d^ +	2.87 ± 0.07^b^ +	1.89 ± 0.09^c^ +	<1.0^d^ +	4.77 ± 0.02^a^ +	4.86 ± 0.01^a^ +
9	<1.0^d^ +	3.76 ± 0.56^b^ +	1.92 ± 0.34^c^ +	<1.0^d^ +	4.94 ± 0.01^a^ +	4.92 ± 0.01^a^ +
12	0.85 ± 0.15^d^ +	3.02 ± 0.21^b^ +	2.15 ± 0.15^c^ +	<1.0^d^ +	<5.0^a^ +	5.4 ± 0.07^a^ +
15	<1.0^b^ +	<1.0^b^ +	<1.0^b^ +	<1.0^b^ +	4.24 ± 0.24^a^ +	–
18	<1.0^a^ +	<1.3^a^ +	<1.0^a^ +	<1.0^a^ +	−	−
21	2.26 ± 0.2^a^ +	−	<1.0^b^ +	<1.0^b^ +	−	−
24	−	−	<1.0 +	−	−	−
27	−	−	<1.0 +	−	−	−
30	−	−	<1.0 +	−	−	−
33	−	−	−	−	−	−

The aerobic plate count was expressed as mean ± standard deviation; if the plates coated by the sample solution and its dilutions were no colony growth, the result was expressed as < 1.0. “+” means that no *S. aureus* was detected. “−” means that each group reaches the end of the shelf life due to the detection of *S. aureus*, and no subsequent experiments are carried out. Lowercase letters indicate differences in each row of data.

Different sterilization methods may have a significant impact on the total aerobic bacteria count (*p* < 0.05). In the initial three days of storage, the aerobic bacteria count in the boiling method group increased to some extent, possibly because the boiling method did not completely eliminate spores. Starting from the 3rd day of storage, the aerobic bacteria count began to decrease and dropped to less than 1.0 lg CFU/g on the 6th, 9th, 12th, 15th, and 18th day of storage. This reduction may be attributed to the inhibitory effects of organic acids, antimicrobial peptides, or bactericidal proteins produced by *L. delbrueckii* spp. Bulgaricus during the fermentation of pork (22). After 21 days of storage, the presence of *S. aureus* was detected, indicating the end of the product’s shelf life. The change in the trend of the aerobic bacteria count in the high-temperature steam sterilized group resembled that of the boiling group. Determinations were halted on the 24th day of storage upon detecting *S. aureus*, signifying the conclusion of the product’s shelf life.

On the 30th day of storage, the total aerobic bacteria count of fermented pork jerky treated with medium-temperature steam sterilization was below 1.0 lg CFU/g. Within the initial 12 days of storage, the total aerobic bacteria count increased, likely due to the proliferation of microorganisms formed from bacterial spores that had not been completely eradicated. Subsequently, there was a decline, potentially because of the accumulation of antibacterial substances generated during the fermentation process, which restricted microbial growth. Additionally, *S. aureus* was detected on the 33rd day of storage. Therefore, fermented pork jerky subjected to medium-temperature steam sterilization exhibited a longer shelf life (extended by 9 days) compared to products treated with high-temperature steam sterilization. The reason behind this difference may be attributed to the application of high temperature and pressure in the latter treatment, leading to microscopic cracks in the packaging material (23). This, in turn, affected its barrier properties by increasing oxygen permeability, consequently promoting microbial growth in the fermented pork (24).

However, the pasteurized group exhibited a higher total aerobic bacteria count during storage, suggesting weaker antibacterial effectiveness compared to the other methods. Starting on the 18th day of storage, determinations were halted due to the detection of *S. aureus*, signifying the conclusion of the product’s shelf life. Ultraviolet sterilization was found to be unsuitable for the sterilization of fermented pork jerky. During the initial stages of storage, the total aerobic bacteria count was relatively high (4.39 ± 0.03 lg CFU/g), and *S. aureus* was detected on the 18th day of storage, signifying the end of the product’s shelf life. Additionally, the ultrasonic sterilization method exhibited a weaker sterilization effect on fermented pork jerky. On the 12th day of storage, the total aerobic bacteria count measured 5.4 ± 0.07 lg CFU/g, failing to meet national standards and thus resulting in the end of the shelf life. In conclusion, the medium-temperature steam method exhibited the most significant impact on sterilizing fermented pork jerky, as it resulted in the highest inhibition of the aerobic plate count.

### 3.2. Changes in total volatile basic nitrogen (TVB-N) during storage of fermented pork jerky treated by different sterilization methods

**Figure 1:**
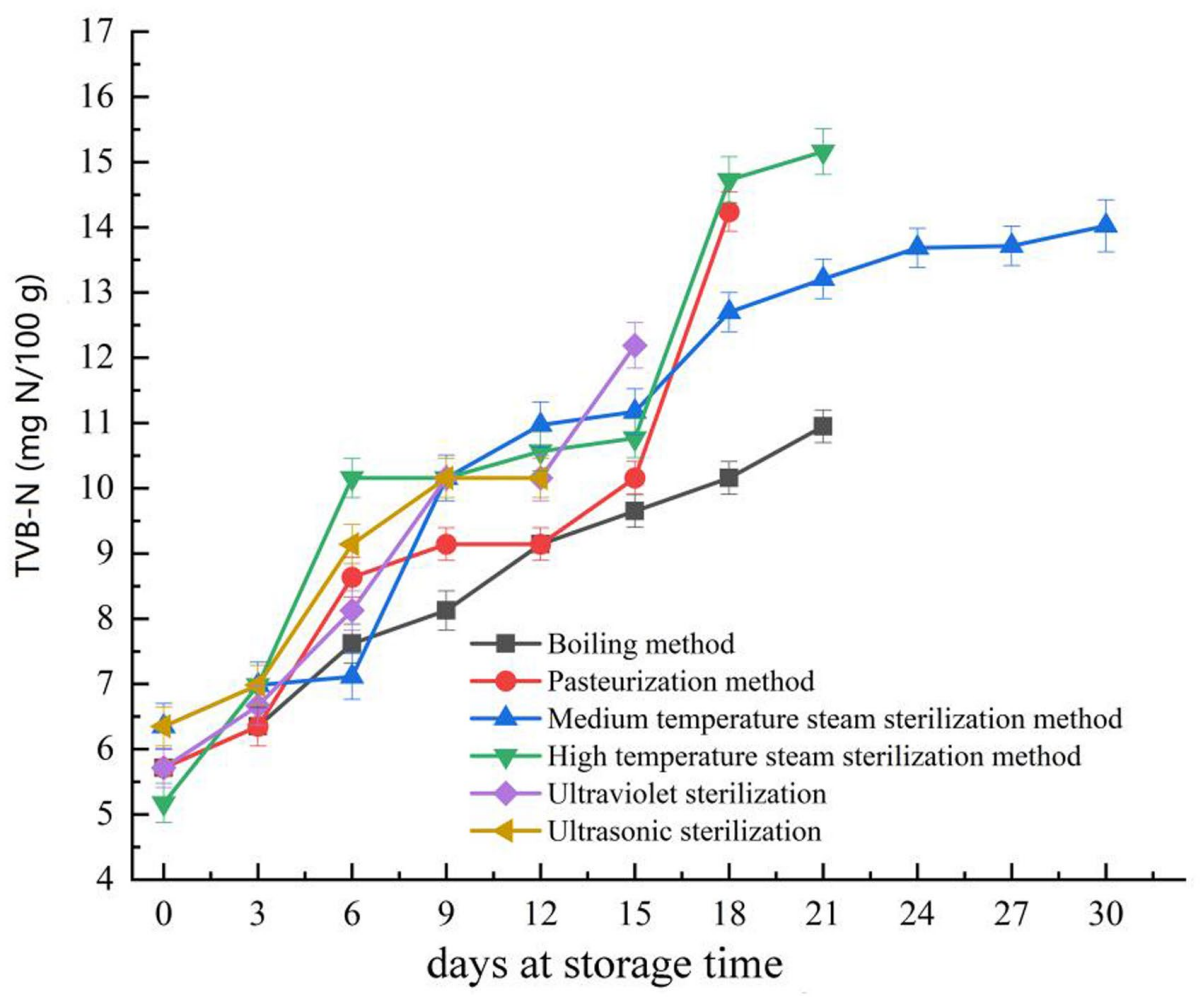
Changes in total volatile basic nitrogen (TVB-N) during storage of pork jerky treated by different sterilization methods.

TVB-N is commonly used as an evaluation standard to determine the quality and shelf life of meat products (25). According to the Chinese national standard “National Food Safety Standards for Fresh (Frozen) Livestock and Poultry Products GB 2707–2016,” (26) the TVB-N content in pork jerky should not exceed 15 milligrams per 100 grams. As seen in Figure 1, the TVB-N levels in fermented pork jerky, treated with six different sterilization methods, increased with the extension of storage time, similar to the findings of Cui’s study on the quality changes in fermented rabbit sausages during storage (27). The increase in TVB-N content during meat storage aligns with other biomarkers of spoilage (such as duration, temperature, packaging, etc.) (28). This increase in TVB-N may be attributed to the degradation of proteins and non-protein nitrogen compounds (29).

### 3.3. Changes in thiobarbituric acid reactive substances (TBARS) during storage of fermented pork jerky treated by different sterilization methods

**Figure 2:**
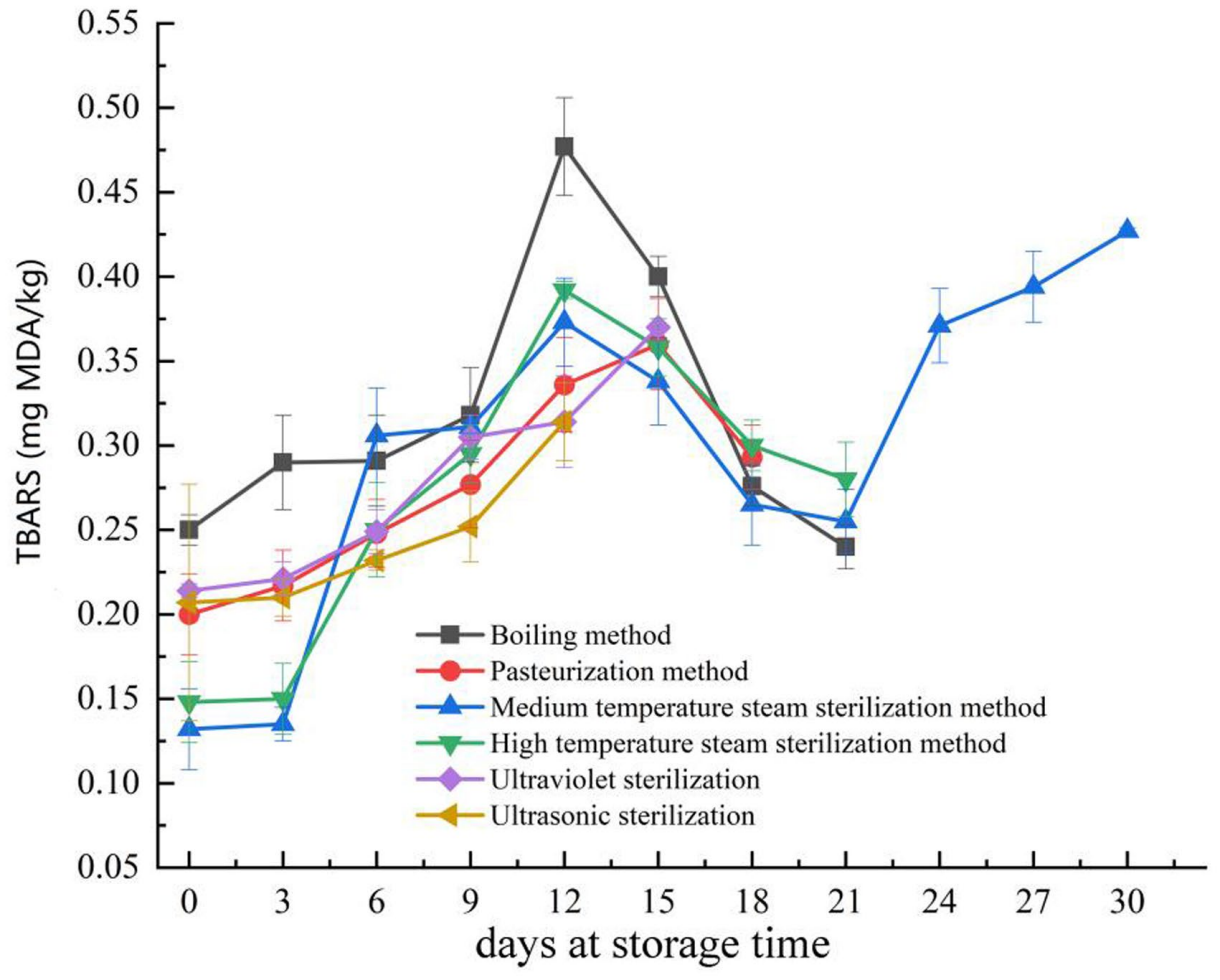
Changes in thiobarbituric acid reactive substances (TBARS) during storage of pork jerky treated by different sterilization methods.

TBARS (Thiobarbituric Acid Reactive Substances) is one of the typical indicators used to assess lipid oxidation in meat products and serves as an evaluation criterion for meat product quality during storage. Consequently, a higher TBARS value indicates a greater degree of lipid oxidation, signifying lower product quality (30, 31). When the TBARS value exceeds 1.0 mg MDA/kg, it can result in the detection of an odor due to product fat oxidation (32). As shown in Figure 2, all six sterilization methods maintained relatively low TBARS values during storage and did not exceed the TBARS limit from the initial stage up to the 30th day of storage. Pork jerky treated with the medium-temperature steam sterilization method exhibited the lowest TBARS values in the first 3 days. Between days 3 and 12, pork jerky treated with the ultrasonic sterilization method had the lowest TBARS values, but its shelf life was shorter due to the detection of *S. aureus*. From days 12 to 21, the TBARS values of pork jerky treated with the medium-temperature steam sterilization method were generally lower than those treated with boiling, high-temperature steam sterilization, and pasteurization methods. During the initial 21 days, the six sterilization methods were ranked based on the maximum TBARS values, with the quality of pork jerky listed in descending order as follows: ultrasonic sterilization method, pasteurization method, ultraviolet sterilization method, medium-temperature steam sterilization method, high-temperature steam sterilization method, and boiling method. Changes in TBARS were associated with the rate of formation and degradation of lipid oxidation products (33). In the initial storage stage, the increase in TBARS may be attributed to the accumulation of lipid oxidation products, such as MDA (Malondialdehyde) (34). In the mid-storage stage, TBARS decreased as malondialdehyde further oxidized into carboxylic acids and organic alcohols (35).

### 3.4. Changes in pH during storage of fermented pork jerky treated by different sterilization methods

**Figure 3:**
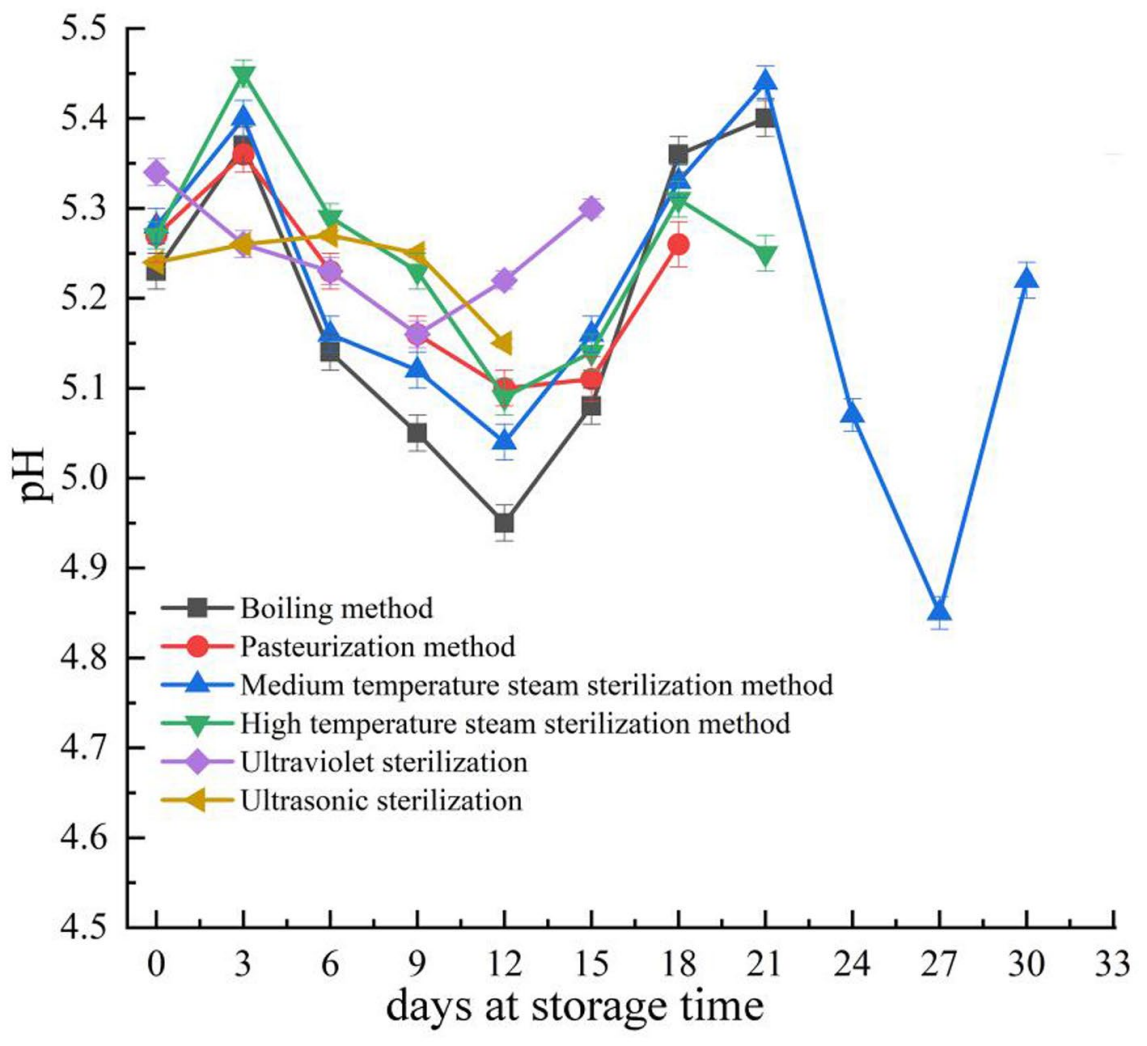
Changes in pH during storage of pork jerky treated by different sterilization methods. The pH of fermented pork jerky in each group was not determined after the end of the shelf life.

A lower pH value can inhibit or delay the microbial degradation of various meat products (36), so pH value needs to be considered during the production of meat jerky (37). Fermented meat products require a rapid decrease in pH to below 5.3 within 48 h during fermentation (38). As shown in Figure 3, the initial pH of ultraviolet sterilization is higher than 5.3. During storage, the pH of fermented pork jerky treated by boiling, pasteurization, medium-temperature steam sterilization, and high-temperature steam sterilization fluctuated significantly. It decreased initially with storage time (up to the 9th day) and then increased in fermented pork jerky sterilized by the ultraviolet method. In contrast, it gradually increased with storage time (up to the 6th day) and then decreased in fermented pork jerky treated by the ultrasonic method, with pH levels consistently below 5.3. Changes in the pH of fermented pork jerky during storage are primarily influenced by internal physical and chemical reactions but are also impacted by the propagation and growth of microorganisms (39). Generally, the accumulation of basic substances (expressed as TVB-N) leads to higher pH values. However, the gradual oxidation and decomposition of fat, along with the growth and reproduction of microorganisms, result in the breakdown of carbohydrates into acidic components, thus lowering the pH (40). In the later stages of storage, the aerobic plate count of pork jerky treated by the medium-temperature sterilization method decreased significantly. At this point, the rate of acidity decrease may be slower than the rate of protein decomposition, which produces alkaline substances such as ammonia and trimethylamine, causing the pH to rise (41).

### 3.5. Changes in Aw during storage of fermented pork jerky treated by different sterilization methods

**Figure 4:**
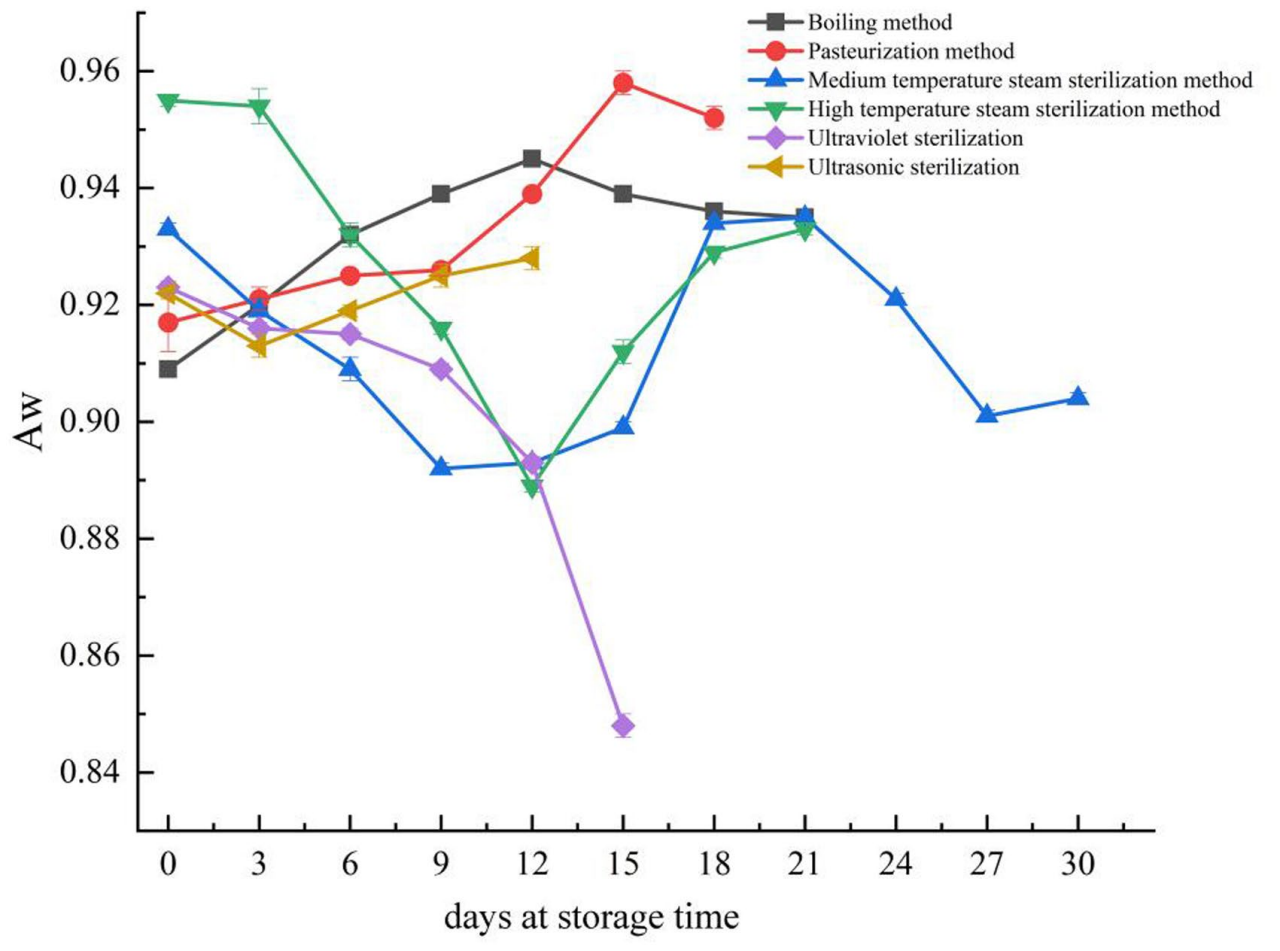
Changes in Aw during storage of pork jerky treated by different sterilization methods. Aw is not determined after the end of fermented pork jerky shelf life in each group.

Reducing water activity (Aw) can prevent microbial growth, which is why Aw is an important factor affecting bacterial growth and the shelf-life stability of jerky (42). As shown in Figure 4, during the storage of fermented pork jerky, only the Aw of the ultraviolet sterilization group continuously decreased. The Aw of fermented pork jerky treated by boiling and pasteurization initially increased and then decreased. In contrast, the Aw of fermented pork jerky treated by high-temperature steam sterilization and medium-temperature steam sterilization decreased initially and then increased, with the high-temperature steam sterilization group having a significantly higher initial Aw than the other groups. The Aw of fermented pork jerky treated by all six sterilization methods was higher than 0.86. According to a recent report, *S. aureus* may grow when Aw exceeds 0.86, which could explain the detection of *S. aureus* in some samples (43). The changes in Aw may be attributed to the substances produced by the decomposition of proteins and fats, such as amino acids and fatty acids, which alter the osmotic pressure of the mixed system and consequently affect the Aw of fermented pork jerky (44, 45).

### 3.6. Sensory scores during storage of fermented pork jerky treated by different sterilization methods

Table 4 illustrates that as the storage time increased, the sensory score exhibited a gradual decline. Both the storage time and sterilization method significantly influenced sensory parameters (*p* < 0.05). During the initial stage, fermented pork jerky treated with the boiling method achieved the highest sensory score, suggesting a minimal impact on the product’s sensory quality. Conversely, fermented pork jerky subjected to high-temperature steam sterilization received the lowest sensory score, possibly attributable to the high-temperature sterilization process damaging the organizational structure of the fermented pork jerky and affecting its flavor and taste (46). Fermented pork jerky treated with the medium-temperature steam sterilization method also received a high sensory score in the initial stage, and the sensory evaluation remained acceptable on the 30th day of storage. Therefore, the medium-temperature steam sterilization method proved to be more suitable for processing fermented pork jerky.

**Table 4:** Sensory scores during storage of pork jerky treated by different sterilization methods.

Storage time (days)	Sterilization method					
	Boiling method	Pasteurization method	Medium temperature steam sterilization method	High temperature steam sterilization method	Ultraviolet sterilization method	Ultrasonic sterilization method
0	93.3 ± 0.5^a^	86.7 ± 0.3^c^	87.7 ± 0.7^c^	81.7 ± 0.7^d^	88.6 ± 0.5^b^	87.9 ± 0.2^c^
3	89.0 ± 0.7^a^	82.3 ± 0.4^c^	86.0 ± 0.5^b^	79.0 ± 0.4^d^	85.8 ± 0.4^b^	86.8 ± 0.5^b^
6	87.3 ± 0.4^a^	82.0 ± 0.4^c^	84.7 ± 0.4^b^	76.3 ± 0.5^d^	82.7 ± 0.6^c^	85.5 ± 0.5^b^
9	85.7 ± 0.5^a^	81.7 ± 0.5^b^	81.3 ± 0.5^b^	72.7 ± 0.5^d^	80.6 ± 0.7^c^	80.7 ± 0.4^c^
12	83.6 ± 0.5^a^	79.3 ± 0.6^b^	80.6 ± 0.6^b^	71.9 ± 0.6^c^	78.7 ± 0.6^b^	76.9 ± 0.3^c^
15	81.7 ± 0.5^a^	78.4 ± 0.6^b^	79.7 ± 0.4^a^	70.2 ± 0.5^d^	74.6 ± 0.4^c^	–
18	80.3 ± 0.6^a^	78.5 ± 0.5^b^	78.3 ± 0.5^b^	69.2 ± 0.5^c^	–	–
21	76.4 ± 0.6^a^	–	76.5 ± 0.5^a^	68.8 ± 0.6^b^	–	–
24	–	–	74.6 ± 0.4	–	–	–
27	–	–	73.9 ± 0.4	–	–	–
30	–	–	70.4 ± 0.3	–	–	–
33	–	–	–	–	–	–

“–” means that sensory evaluation is no longer measured after the end of the shelf life of fermented pork in each group. Lowercase letters indicate differences in each row of data.

## 4. Conclusion

This study primarily investigated the effects of various sterilization methods, including boiling, pasteurization, medium-temperature steam sterilization, high-temperature steam sterilization, ultrasonic sterilization, and ultraviolet sterilization, on the shelf life and physicochemical characteristics of vacuum-packaged fermented pork jerky. The results revealed that medium-temperature steam sterilization resulted in the longest shelf life for fermented pork jerky, extending up to 30 days. Considering both physicochemical indicators and sensory evaluations, medium-temperature steam sterilization proved to be the most suitable method for processing fermented pork jerky. Therefore, this experiment identified medium-temperature steam sterilization as the most favorable sterilization method for fermented pork jerky intended for storage, providing valuable insights for the industrial production of fermented pork jerky.

## Data availability statement

The original contributions presented in the study are included in the article/supplementary material, further inquiries can be directed to the corresponding authors.

## Author contributions

CZ: conceptualization (lead) and formal analysis (lead). JD: software (lead) and writing-original draft (lead). FC: writing-review editing (equal). ZZ: resources (lead) and supervision (lead). XZ: formal analysis (equal) and methodology (supporting). All authors contributed to the article and approved the submitted version.

## References

[1] Karaçil M Nilüfer A. Dünyada üretilen fermente ürünler: tarihsel süreç ve sağlık ile ilişkileri. Uludağ Üniversitesi Ziraat Fakültesi Dergisi. (2013) 27:163–74.

[2] Chen F Zhao C Feng J Zhao Y. Optimization conditions for the prolonged storage of fermented pork jerky using *Lactobacillus bulgaricus* as a starter culture. J Food Proc Preserv. (2020) 45:e14921. doi: 10.1111/JFPP.14921

[3] Chen FF Zhao CQ Wang SC. Optimization of conditions for prolonging shelf life of fermented pork jerky by *Lactobacillus acidophilus*. China Condiment. (2020) 45:5–9. doi: 10.1111/jfpp.14921

[4] Chen FF Zhao CQ Zhao Y. Research on prolonging storage period of pork jerky fermented by *Pediococcus acidilactici*. China Condiment. (2020) 45:63–7.

[5] Liu Y Hou ZZ. The development of new sterilization technology. Modern Food. (2018) 131-134. doi: 10.16736/j.cnki.cn41-1434/ts.2018.03.041

[6] Chouliara E Karatapanis A Savvaidis IN Kontominas MG. Combined effect of oregano essential oil and modified atmosphere packaging on shelf-life extension of fresh chicken breast meat, stored at 4C. Food Microbiol. (2007) 24:607–17. doi: 10.1016/j.fm.2006.12.005, PMID: 17418312

[7] Jiang KX Ma LQ. Technology of Cold Sterilization and its Application in the food processing. China Food Safety. (2020) 17:37.

[8] Zhao B Ren L Zhang JC. Effect of different sterilization methods on smoked meat. Meat Res. (2012) 10:13–7.

[9] Zhang JK Bao QP Sun ZF. Research progress on new sterilization technology in food processing. J Food Saf Qual. (2017) 8:3099–103.

[10] Wang M Li TZ Lei J. Effects of different sterilization methods on quality of cooked meat products. Food Industry. (2016) 37:54–8.

[11] James RM James M Farmer DR. Method and apparatus for steam pasteurization of meat:US19980183272[P]. US6291003B1[2023-10-10].

[12] National food safety standard food microbiological-the aerobic plate count for determination GB 4789.2-2016. (2016).

[13] National food safety standard food microbiological-the coliforms count for determination GB 4789.3-2016. (2016).

[14] National food safety standard food microbiological-the *Staphylococcus aureus* test for detection GB 4789.10-2016. (2016).

[15] National food safety standard food microbiological-the *Shigella* test for detection GB 4789.5-2012. (2012).

[16] National food safety standard food microbiological-the *Salmonella* test for detection GB4789.4-2016. (2016).

[17] Xiaozhen Y Jinbiao Z Yongxu C. The evaluation of the three edible tissues of dead adult Chinese mitten crabs (*Eriocheir sinensis*) freshness in harvest season, based on the analysis of TVBN and biogenic amine. Springerplus. (2016) 5:1906. doi: 10.1186/s40064-016-3434-4, PMID: 27867813 PMC5095103

[18] Reitznerova A Sulekova M Nagy J Marcincak S Semjon B Certik M. Lipid peroxidation process in meat and meat products: a comparison study of malondialdehyde determination between modified 2-Thiobarbituric acid spectrophotometric method and reverse-phase high-performance liquid chromatography. Molecules. (2017) 22:1988. doi: 10.3390/molecules22111988, PMID: 29144423 PMC6150165

[19] Zhang S. Food Alalysis. Beijing, China: China Light Industry Press (2005).

[20] K.V. To Zhang X Shao W Schilling MW. The effects of dry-cured ham initial water activity on Tyrophagus putrescentiae infestations. J Stored Prod Res. (2020) 87:101609. doi: 10.1016/j.jspr.2020.101609

[21] Wang XH Li JX Tan ML. Effect of mixed starter cultures on quality of fermented pork jerky. Sci Technol Food Ind. (2015) 17:165–9. doi: 10.13386/j.issn1002-0306.2015.17.025

[22] Tian T Zhan RQ Zhang YL. Study on safety improvement of Cantonese sausage by microbial fermentation technology. China Condiment. (2019) 44:26–30.

[23] Guo ZL Han L Yu QL Lin L. Effect of a sea buckthorn pomace extract-esterified potato starch film on the quality and spoilage bacteria of beef jerky sold in supermarket. Food Chem. (2020) 326:127001. doi: 10.1016/j.foodchem.2020.127001, PMID: 32416417

[24] Tang YL Zhao W Lu LX. Sterilization processing under ultra high pressure on the stability of packaging materials. Packag Eng. (2010) 23:10–2. doi: 10.19554/j.cnki.1001-3563.2010.23.005

[25] Zhu Y Ma L Yang H Xiao Y Xiong YL. Super-chilling (−0.7°C) with high-CO2 packaging inhibits biochemical changes of microbial origin in catfish (*Clarias gariepinus*) muscle during storage. Food Chem. (2016) 206:182–90. doi: 10.1016/j.foodchem.2016.03.053, PMID: 27041314

[26] National food safety standard-fresh (frozen) livestock and poultry products GB 2707-2016. (2016).

[27] Cui G J. (2017). Study on processing Technology of Fermented Rabbit Sausage and the variation of quality during storage. Southwest University. Available at: http://gffiy28995338bdc041dasw05qc6bcvqwc60kk.fffb.suse.cwkeji.cn:999/KCMS/detail/detail.aspx?dbname=CMFD201801&filename=1017846887.nh

[28] El Din A Alaa B Holman Benjamin WB Giteru SG Hopkins DL. Total volatile basic nitrogen (TVB-N) and its role in meat spoilage: a review. Trends Food Sci Tech. (2021) 109:280–302. doi: 10.1016/J.TIFS.2021.01.006

[29] Heydari-Majd M Ghanbarzadeh B Shahidi-Noghabi M Hosseini M. A new active nanocomposite film based on PLA/ZnO nanoparticle/essential oils for the preservation of refrigerated *Otolithes ruber* fillets. Food Packag Shelf Life. (2019) 19:94–103. doi: 10.1016/j.fpsl.2018.12.002

[30] Manhani MR Nicoletti MA Barretto ACDS Jesus GRD Camila Munhoz C Abreu GRD. Antioxidant action of rosemary and oregano extract in pre-cooked meat hamburger. Food Nutr Sci. (2018) 9:806–17. doi: 10.4236/fns.2018.97060

[31] Pignoli G Bou R Rodriguez-Estrada MT Decker EA. Suitability of saturated aldehydes as lipid oxidation markers in washed Turkey meat. Meat Sci. (2009) 83:412–6. doi: 10.1016/j.meatsci.2009.06.019, PMID: 20416697

[32] Mccoy G Houser T Stroda S Boyle E. Estimating the shelf life of whole muscle Bison jerky using accelerated storage conditions. Meat Muscle Biol. (2010). doi: 10.2217/51rmc2016.070

[33] Yang H-S Hwang Y-H Joo S-T Park G-B. The physicochemical and microbiological characteristics of pork jerky in comparison to beef jerky. Meat Science. (2009) 82:289–94. doi: 10.1016/j.meatsci.2009.01.029, PMID: 20416733

[34] Jin SK Kim IS Hah KH Lyou HJ Park KH Lee JR. Quality characteristics of vacuum packaged fermented pork with soy sauce, red pepper and soybean paste seasoning during storage. J Anim Sci Technol. (2005) 47:825–36. doi: 10.5187/jast.2005.47.5.825

[35] Verma SP Sahoo J. Improvement in the quality of ground chevon during refrigerated storage by tocopherol acetate preblending. Meat Sci. (2000) 56:403–13. doi: 10.1016/S0309-1740(00)00072-3, PMID: 22062171

[36] Luo Y Zhao L Xu J. Effect of fermentation and postcooking procedure on quality parameters and volatile compounds of beef jerky. Food Sci Nutr. (2020) 8:2316–26. doi: 10.1002/fsn3.1515, PMID: 32405389 PMC7215205

[37] Bowser TJ Scott FR Weckler PR Kowalski SJ. Optimizing jerky drying time with minimal product impact. Open Food Sci J. (2009) 3:79–83. doi: 10.2174/1874256400903010079

[38] Porto-Fett ACS Espuña E Shane LE Shoyer BA McGeary L Vinyard BT. Viability of Shiga toxin-producing *Escherichia coli*, *Salmonella* spp., and *Listeria monocytogenes* during preparation and storage of Fuet, a traditional dry-cured Spanish pork sausage. J Food Prot. (2022). 85:879–89. doi: 10.4315/JFP-21-356 35294002

[39] Zhang DS Gen LL Shi WD. Experimental investigation on pressure fluctuation and vibration in axial-flow pump model. Trans Chin Soc Agric. (2015) 46:66–72. doi: 10.6041/j.issn.1000-1298.2015.06.010

[40] Zhou LD Ai MM Ling ZT. Study on quality change and shelf of Guangdong style salt baked chicken storage in 4°C. Food Mach. (2018) 34:115–20.

[41] Fuentes-Amaya LF Munyard S Fernandez-Piquer J Howieson J. Sensory, microbiological and chemical changes in vacuum-packaged blue spotted emperor (Lethrinus sp), Saddletail snapper (*Lutjanus malabaricus*), crimson snapper (*Lutjanus erythropterus*), barramundi (*Lates calcarifer*) and Atlantic Salmon (*Salmo salar*) fillets stored at 4 degrees C. Food Sci Nutr. (2016) 4:479–89. doi: 10.1002/fsn3.309, PMID: 27247777 PMC4867767

[42] Kocatepe D Turan H Altan CO. Effect of the vacuum packaging on the shelf life of Lakerda. Int J Food Sci. (2014) 3. doi: 10.19070/2326-3350-1400029 26337982

[43] Jay JM Loessner MJ Golden DA. Modern food microbiology[J] Chapman Hall (1996).

[44] Chen WS Liu DC Chen MT. Determination of quality changes throughout processing steps in Chinese-style pork jerky. Asian Australas J Anim Sci. (2004) 17:700–4. doi: 10.5713/ajas.2004.700

[45] Shang X Cao CA Zhang YM. Effects of different type of packaging bags on quality changes of Western bacon during chilling storage. Food Res Dev. (2019) 5:28–34.

[46] Barbosa-Canovas GV Medina-Meza I Candogan K Bermudez-Aguirre D. Advanced retorting, microwave assisted thermal sterilization (mats), and pressure assisted thermal sterilization (pats) to process meat products. Meat Sci. (2014) 98:420–34. doi: 10.1016/j.meatsci.2014.06.027, PMID: 25060584

